# PET/CT imaging of giant primary pulmonary meningioma: a case report and literature review

**DOI:** 10.1186/s13019-023-02276-4

**Published:** 2023-05-03

**Authors:** Yawen Feng, Peng Wang, Yufei Liu, Wenli Dai

**Affiliations:** 1 Department of Nuclear Medicine, the First College of Clinical Medical Science, China Three Gorges University, Yichang, 443003 Hubei China; 2 Department of Pathology, The First College of Clinical Medical Science of China, Three Gorges University and Yichang Central People’s Hospital, No. 183, Yiling Avenue, Yichang, 443000 China

**Keywords:** Primary pulmonary meningioma, ^18^F-fluorodeoxyglucose, PET/CT, Case report

## Abstract

**Background:**

An ectopic meningioma, such as a primary pulmonary meningioma (PPM), is a rare type of tumor that primarily originates outside of the central nervous system. The most common presentation of PPM is isolated pulmonary nodules or masses, and most of them are benign. Only sporadic cases have been reported. This case reported a giant primary pulmonary meningioma and systematically reviewed previously reported cases in the literature.

**Case presentation:**

A 55-year-old female suffered from asthma after activity, chest tightness, and a persistent dry cough for 2 months. Chest computed tomography (CT) showed a huge mass with calcification in the left lower lobe. And positron emission tomography (PET)/CT revealed mild FDG accumulation of the mass. The mass was finally surgically removed and PPM was confirmed according to histopathologic examinations.

**Conclusion:**

PPM is a rare disease with heterogeneity not only in CT features but also in glucose metabolism. FDG uptake levels do not identify benign from malignant, benign PPM may have high FDG uptake and malignant may have low.

## Background

Meningiomas are common tumors of the nervous system originating from arachnoid cells, which often occur intracranially or in the spinal canal, and are mostly benign. Meningiomas occurring in tissues and organs not covered by meninges are called ectopic meningiomas which are very rare tumors that commonly occur in the head and neck or, less frequently, in the skin [1, 2]. Primary meningioma in lung tissue is defined as primary pulmonary meningioma. Since the first case report in 1982 by Kemnitz et al [3], only a small number of cases of PPMs have been reported. With the development of medical imaging disciplines, we can make a preliminary diagnosis or differential diagnosis of PPM through imaging. Herein, we report a case of a giant PPM diagnosed with ^18^ F-FDG PET/CT imaging and a review of the literature on previous instances of ectopic meningioma of the thoracic cavity diagnosed with ^18^ F-FDG PET/CT imaging.

## Case presentation

**Fig. 1:**
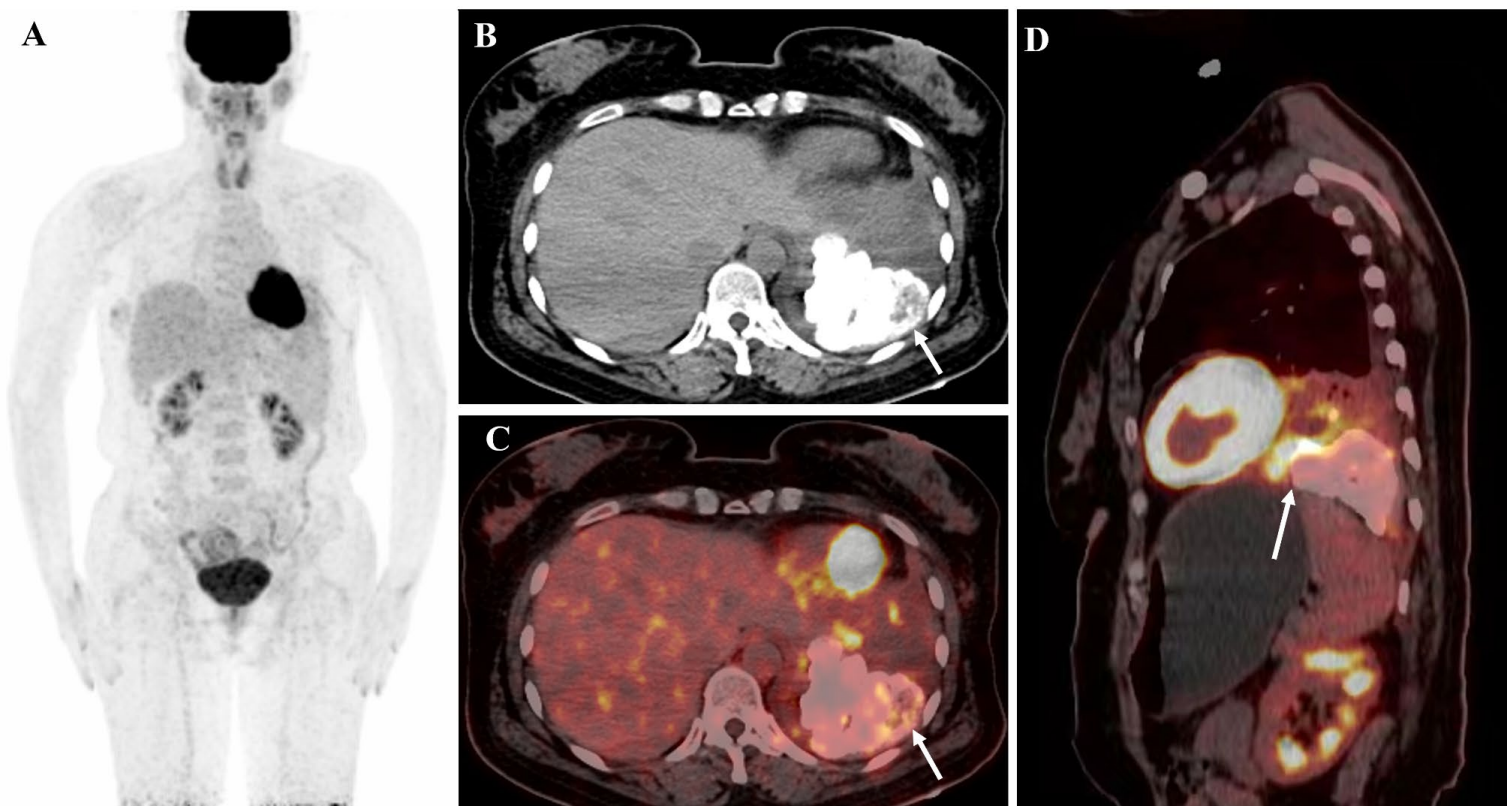
The maximum intensity projection PET Image (A) in the whole body. Transverse chest CT showing a huge tumor with massive calcification (B), and fusion PET/CT (C) images demonstrating a huge tumor with mild high metabolic activity (SUVmax 4.4) and the size of it about 76 mm × 70 mm × 59 mm. Sagittal PET/CT image (D) demonstrating the site of the highest metabolism (SUVmax 8.1)

**Fig. 2:**
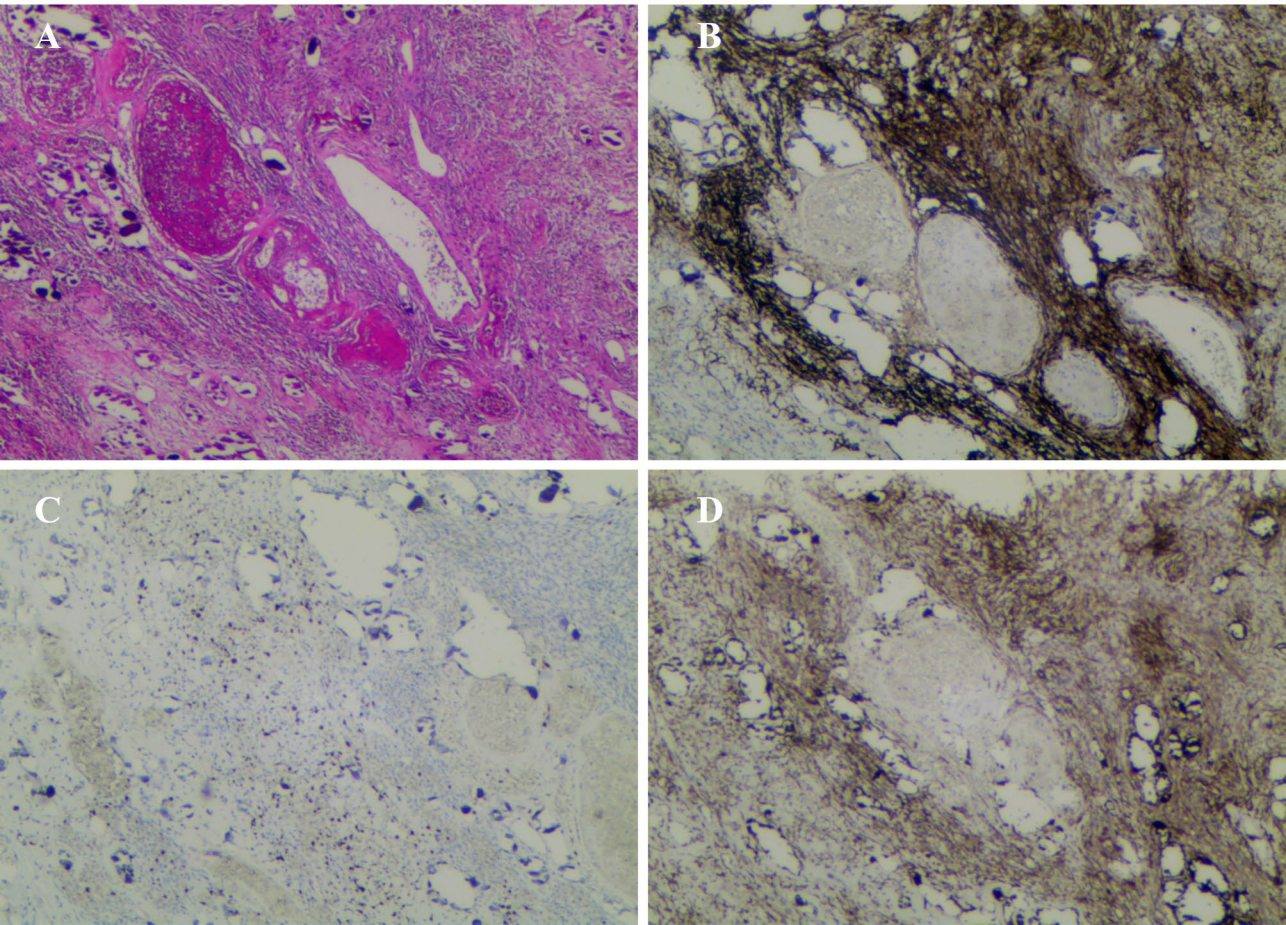
Pathological examination. Hematoxylin-eosin Stain (A), Original Magnification 100×.The tumor cells stained positive for EMA (B, 100×) and SSTR2 (D, 100×), and Ki-67 index was about 20% (C, 100×). These findings are supporting the diagnosis of pulmonary meningioma

A 55-year-old female patient was admitted to our hospital complaining of asthma after activity, chest tightness, and a persistent dry cough for 2 months. A physical examination of the left side of the chest revealed decreased respiratory motion, decreased breath sounds, and turbid percussion in the left side of the chest. After admission, routine blood tests, lung cancer tumor markers and node antacid staining did not show any significant abnormalities. Pleural fluid cytopathological examination showed a small number of lymphocytes, macrophages, mesothelial cells, and no malignant tumor cells. A chest computed tomography (CT) performed a lobulated pulmonary mass with calcification in the left lower lobe, and adjacent pleural hypertrophy and adhesions. The patient underwent the ^18^ F-FDG PET/CT examination to clarify the diagnosis. On ^18^ F-FDG PET imaging (Fig. 1), the standardized uptake value (SUV) of the mass increased unevenly, with SUVmax from 4.4 to 8.1. The size of the mass is about 76 mm × 70 mm × 59 mm. And without lesions identified in other locations in his body (including nervous system and spine). The patient’s diagnosis and treatment process showed in the Table 1. Due to massive calcified lesions and mild high metabolic activity, the nuclear medicine physician considered the mass to be benign. For further diagnosis and treatment, the patient received a surgery. Intraoperative exploration revealed an 8 cm diameter calcified mass between the left lower lung and the diaphragm with adhesions to the surrounding lung tissue and the diaphragm. Subsequently, the patient underwent left thoracic adhesion, mass excision, wedge resection of the left lower lung lobe and intraoperative frozen pathological examination. Gross examination revealed a grayish-white mass measuring 9.5cmx8.4cmx5.3 cm. Microscopic examination showed mainly spindle cells, with mild cell morphology. The tumor involved the pleura with fusiform nests of cells arranged in fascicles or whorls and extensive calcification. Immunohistochemistry showed positivity for epithelial membrane antigen (EMA), progesterone receptor (PR), somatostatin receptor 2 (SSTR2), CD34, and S-100, and negativity for Desmin, STAT6, ALK(1A4), and SMA. The Ki-67 index was about 20% positive. These morphological and immunohistochemical features were suggestive of a benign PPM (WHO 1 grade) (Fig. 2).

**Table 1:** Patient examination and treatment procedure

Items	Detailed information
**Laboratory Examination**	
Hematological	
WBC	7.27 × 10^9/L
RBC	4.16 × 10^12/L
Blood platelet	284 × 10^9/L
Lung cancer tumor markers	
CEA	1.4ng/ml
NSE	14.1ng/ml
Cyfra21-1	1.2ng/ml
ProGRP	32.9pg/ml
SCC	0.3ng/ml
Examinations of pleural fluid	
TP	66.36 g/L
ALB	31.28 g/L
LDH	1823IU/L
T-SPOT	(-)
Acid-fast staining	(-)
**Imaging examinations**	
CT of the chest	A lobulated mass in the lower lobe of the left lung with massive calcification, measuring about 76 mm × 70 mm × 59 mm.
^18^ F-FDG PET/CT	Uneven metabolism of the masses, with SUVmax from 4.4 to 8.1.
**Treatment**	Surgical operation
**Pathological results**	Meningioma (Benign, WHO I Grade)
**Follow up**	No recurrent lesions were found in the operated area at the 1-year postoperative review CT.

WBC: white blood cell; RBC: red blood cell; CEA: carcinoembryonic antigen; NSE: euron specific enolase; Cyfra21-1: cytokeratin 19 fragment; ProGRP: pro-gastrin-releasing peptide; SCC: squamous cell carcinoma; TP: Total protein; ALB: albumin; CT: computed tomography; ^18^ F-FDG PET: 18 F-fluorodeoxyglucose positron emission tomography.

## Discussion

The incidence of ectopic meningioma is very rare (2%) and can be located in various anatomical sites such as the scalp, maxillary sinus, sinus lung, parotid gland, skin, and peripheral nerves [4]. The pathogenesis of PPM is still unclear, and it is generally believed that ectopic meningioma may derive from pluripotential subpleural mesenchymal cells or heterotopic embryonic rests of arachnoid cells [1, 2]. Most patients with PPM do not have significant symptoms, while some patients have respiratory. Clinical symptoms may be related to the position of the lesion and the size of the lesion [5]. In this case, the patient had chest tightness, shortness of breath, and cough, probably due to the huge mass adjacent to the pleura. Most PPMs were benign, and only a few cases are malignant [6–10]. Patients with benign PPM have a better prognosis and generally do not recur or metastasize distantly.

Imaging characteristics can be distinguished to some degree from other types of tumors. On chest CT scans, PPMs usually appear as isolated, round, solid, well-defined nodules or masses, with or without calcification. But a few cases present with ground-glass nodules or multiple cystic lesions [11, 12]. The size of benign PPMs ranged from 0.4 to 6 cm in diameter (median: 2 cm). The size of malignant PPMs ranged from 1.5 to 15 cm in diameter (median: 6.4 cm) [5]. Although the average diameter of malignant PPMs is longer than benign PPMs, it is not possible to distinguish between benign and malignant based on the size of the tumor alone. As we reported a huge PPM, measuring 9.5cmx8.4cmx5.3 cm, but the pathology results confirmed that it was a benign tumor.

To further learn the characteristics of ^18^ F-FDG PET/CT images of PPMs, we performed a systematic search of PubMed and web of science using the keywords “primary pulmonary meningioma”, “PET”, “ectopic meningioma”. And finally, we got 11 case reports about primary pulmonary meningioma with complete data of PET/CT and listed them in Table 2. On ^18^ F-FDG PET, PPMs exhibit mild to high metabolic activity, and the average value of SUVmax is 4.36, ranging from 0.6 to 12.9. The lesion with the SUVmax of 0.6 was a well-defined nodular lesion, measuring approximately 1.3 cm × 1.3 cm × 1.5 cm, whose pathological findings were benign PPM [13]. The lesion with SUVmax of 12.9 was also a benign PPM, measuring 1.5 cm, and appeared as a lobulated nodule on CT images [14]. The largest PPM was the one we reported in this article. The size was 9.5 cm x 8.4 cm x 5.3 cm and the SUVmax value was from 4.4 to 6.2, showing mild to moderate uptake of ^18^ F-FDG. Due to the mass having a multitude of calcified lesions and mostly mild FDG uptake, the mass was diagnosed as a benign lesion at the time of initial diagnosis. Therefore, the level of SUVmax alone cannot evaluate the nature of PPMs (benign vs. malignant). The case reported by Andrea Cimini et al [7] also confirmed this view. In their case report, a patient had two PPMs, and the malignant PPM had lower SUVmax values than the benign PPM. Consequently, PPMs are mainly diagnosed by pathology, while imaging medicine can initially distinguish PPMs from other tumors.

**Table 2:** Imaging and PET/CT features of thoracic ectopic meningioma case

NO.	Ref.	Age	gender	Symptom	CT feature	Site	Tumor size (cm)	Contrast-enhanced CT	PET/CT	Histology
1	Danbin Zhang [5]	47	F	None	A lobulated mass with calcification.	LL	6.9 × 5.7 × 6.1	Mild enhancement	SUVmax 4.4	B
2	Maoqing Jiang [13]	70	M	None	A solitary pulmonary nodule with well-defined.	RL	1.3 × 1.3 × 1.5	Mild enhancement	SUVmax 0.6	B
3	CURA [15]	58	F	None	A well-circumscribed mass.	RU	2	N	Metabolic activity	B
4	Meirelles [14]	48	M	None	A lobulated nodule.	RU	1.5	N	SUVmax 12.9	B
5	Incarbone [16]	24	M	None	A solitary pulmonary nodule.	RU	1.4	Mild enhancement	SUVmax 10.14	B
6	Lepanto [17]	60	F	None	A solitary pulmonary nodule.	LU	1.2	N	SUVmax 1.2	B
7	Bae [18]	43	F	None	A solitary pulmonary nodule.	LU	1.7	Well enhancement	SUVmax 2.48	B
8	Gürçay [12]	55	F	Coughing	A solitary ground-glass nodule.	RU	2 × 2 × 1.8	N	SUVmax 1.89	B
9	Oh [19]	54	M	None	Multiple lung nodules	RU	0.96	N	SUVmax 3.1	B
						LU	1.25	N	SUVmax 2.3	B
10	Cimini [7]	80	M	None	A solitary pulmonary nodule.	RU	1.4	N	SUVmax 4.63	B
					A solitary pulmonary nodule.	LR	1.2	Well enhancement	SUVmax 2.46	M
11	BAŞ [20]	57	M	Coughing	A solitary pulmonary nodule.	LL	1.0	N	^18^ F-FDG no uptake	B
12	This report	55	F	Asthma, cough, chest tightness	A lobulated mass with calcification.	LL	9.5 × 8.4 × 5.3	N	SUVmax 6.2	B

F: Female; M: Male; None: No symptoms; B: Benign; M: Malignant; RL: Right lower lobe; RU: Right upper lobe; LL: Left lower lobe; LU: Left upper lobe; N: Not reported.

Benign PPMs can be cured by surgical treatment. Malignant PPMs are prone to recurrence or metastasis hence the prognosis is poor. In general, most PPM have a good prognosis without recurrence or metastasis [16], and the main strategy of treatment is surgical resection [21]. The patient, in our case, received surgery and had been followed up for 9 months without tumor recurrence.

## Conclusion

In this paper, we reported a huge mass in the, with massive calcified lesions, and mild to moderate metabolism, the final diagnosis was benign PPM. We reviewed the literature and found that most of PPMs usually appear as isolated, round, solid, well-defined nodules or masses, with or without calcification on CT scan and exhibit mild to high metabolic activity on PET scan, and the level of SUVmax alone cannot evaluate the nature of PPMs (benign vs. malignant).

## Data Availability

Data sharing is not applicable to this article as no datasets were generated or analysed.

## Abbreviations

^18^F-FDG-PET/CT: 18 F-fluorodeoxyglucose positron-emission tomography-computed tomography
SUVmax: Maximum Standardized Uptake Value
PPM: Primary pulmonary meningioma
CT: computed tomography
F: Female
M: Male
None: No symptoms
B: Benign
M: Malignant
RL: Right lower lobe
RU: Right upper lobe
LL: Left lower lobe
LU: Left upper lobe
N: Not reported.
